# Neoadjuvant therapy-induced immune dynamics and myeloid-associated resistance in advanced head and neck cancer

**DOI:** 10.1038/s41698-025-00954-1

**Published:** 2025-06-07

**Authors:** Alisa Kimura, Junichi Mitsuda, Kanako Yoshimura, Sumiyo Saburi, Nanako Murakami, Nana Sakurai, Koichi Yoshizawa, Hiroki Morimoto, Shigeyuki Mukudai, Hikaru Nagao, Hiroshi Ogi, Saya Shibata, Aya Miyagawa-Hayashino, Eiichi Konishi, Kyoko Itoh, Shigeru Hirano, Takahiro Tsujikawa

**Affiliations:** 1 Department of Otolaryngology–Head and Neck Surgery, Kyoto Prefectural University of Medicine, Kyoto, Japan; 2 Department of Pathology and Applied Neurobiology, Kyoto Prefectural University of Medicine, Kyoto, Japan; 3 SCREEN Holdings Co. Ltd., Kyoto, Japan; 4 Department of Surgical Pathology, Kyoto Prefectural University of Medicine, Kyoto, Japan; 5 Department of Cell, Developmental & Cancer Biology, Oregon Health and Science University, Portland, OR USA

**Keywords:** Head and neck cancer, Cancer microenvironment

## Abstract

Understanding the dynamics of the tumor-immune ecosystem is crucial for advancing neoadjuvant strategies in cancer treatment. This study investigated alterations in the tumor-immune microenvironment related to the response to preoperative combination therapy with paclitaxel, carboplatin, and cetuximab in patients with advanced head and neck squamous cell carcinoma. Thirty patients underwent combination therapy. Biopsy or surgical specimens were obtained before and after treatment. Single-cell-based, 14-marker multiplex immunohistochemistry and image cytometry were employed to assess changes in immune cell densities and profiles. Three distinct immune profiles were identified: hypo-, lymphoid-, and myeloid-inflamed. Significant decreases in tumor volume and increases in CD45^+^ cells and programmed cell death ligand 1 (PD-L1) scores were observed in the hypo- and lymphoid-inflamed groups, whereas the myeloid-inflamed group showed minimal changes. After treatment, increased calreticulin expression in tumor cells, together with increased lymphoid cell lineages, was observed in non-recurrent cases. The myeloid-inflamed group exhibited higher expression of hypoxia inducible factor 1α and zinc finger E-box-binding homeobox 2, suggesting the presence of a hypoxic and metastasis-promoting environment. Spatial analysis revealed that responders had a high infiltration of T cells within tumor cell nests, whereas non-responders had fewer T cells, with β-catenin expression in cancer cells. Upregulated lymphocyte activation gene 3 in the myeloid-inflammation group, and PD-L1 dynamics suggest potential targets for further therapy. These findings highlight the need for targeted neoadjuvant strategies based on immune profiling.

## Introduction

Locally advanced head and neck squamous cell carcinoma (HNSCC) remains a significant challenge because of its high recurrence rate and consequential impact on critical functions, such as swallowing and speech, alongside aesthetic considerations^1^. The shortcomings of the current therapeutic strategies require innovative treatment approaches, including neoadjuvant and combined therapeutics. The tumor-immune microenvironment of HNSCC plays a crucial role in disease progression and response to therapy, because the density, composition, and functional status of lymphoid and myeloid immune cell lineages are closely related to clinical outcomes^2^. Therefore, understanding the impact of existing treatments on the tumor-immune microenvironment and exploring methods to modulate tumor-immune dynamics towards a favorable microenvironment are essential.

Neoadjuvant therapies, including chemotherapy and immunotherapy, are being actively investigated for the treatment of advanced HNSCC to enhance local control and preserve organ function before surgery or chemoradiotherapy. These therapies induce longitudinal changes in the tumor-immune microenvironment^3^. For example, platinum-based medications, taxanes, and cetuximab, which target the epidermal growth factor receptor, increase the frequency of T cells within tumor tissues^4,5^. However, the variability in tumor-immune microenvironmental dynamics during pharmacological interventions based on longitudinal and spatial immune characteristics remains poorly understood. Predicting the dynamics and therapeutic outcomes of baseline tumor-immune characteristics could lead to better control of the microenvironment and more effective neoadjuvant therapies.

This study aimed to elucidate the mechanisms associated with neoadjuvant chemotherapy using paclitaxel, carboplatin, and cetuximab in advanced HNSCC^6^. Using single cell-based, 14-marker multiplex immunohistochemistry (IHC) analysis, we investigated baseline characteristics and longitudinal changes within the tumor-immune microenvironment. Our focus was on understanding the spatiotemporal dynamics of the tumor-immune microenvironment and its correlation with therapeutic response, providing insights into the complexities between tumor cells and immune cell lineages. The present study aimed to enhance our understanding of HNSCC and facilitate effective neoadjuvant strategies.

## Results

### Baseline immune profiles stratify therapeutic effects and immune cell densities following combination therapy with paclitaxel, carboplatin, and cetuximab

**Fig. 1:**
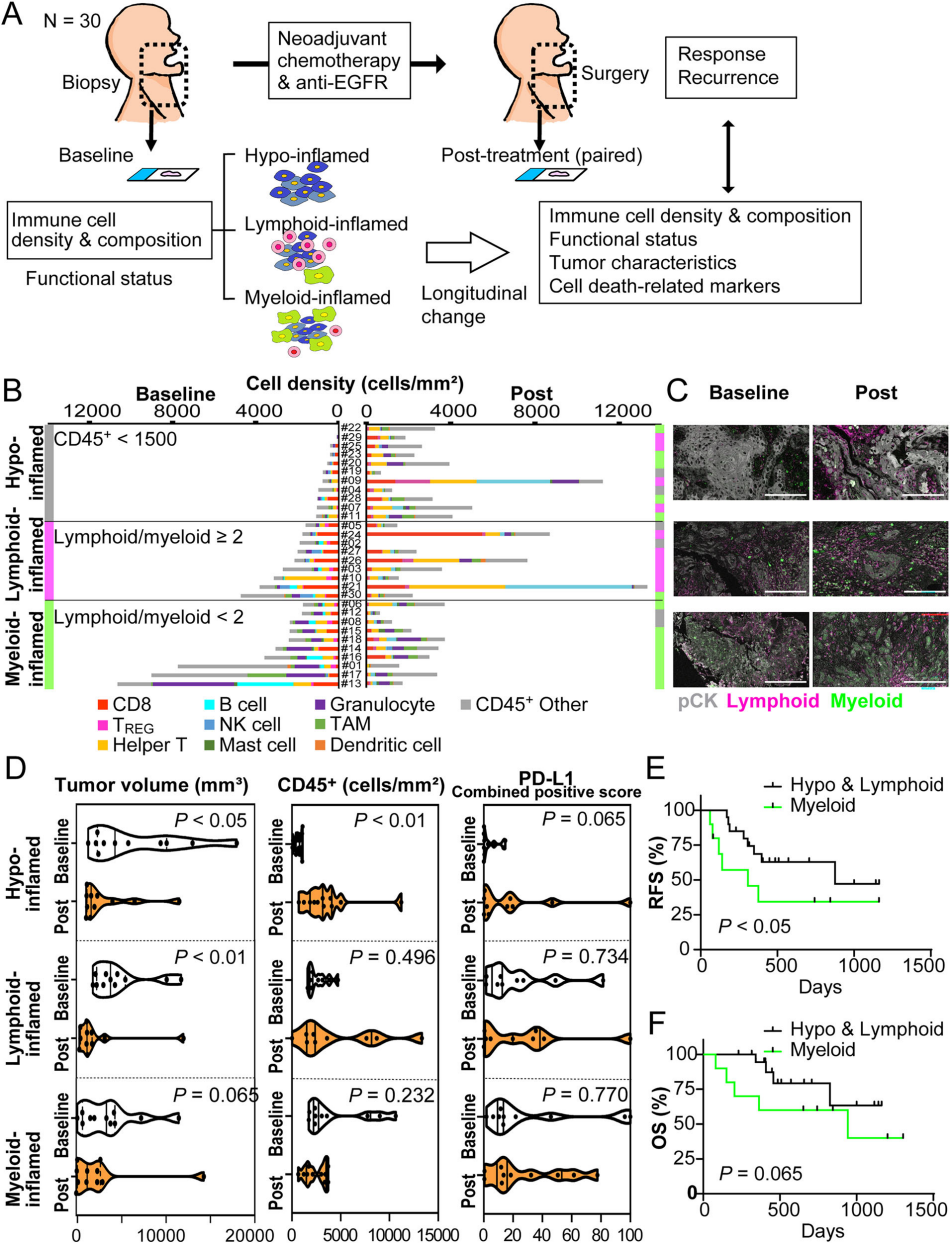
Baseline immune profiles stratify longitudinal change of immune cell densities during chemo-targeted therapy. **A** The study involved biopsy and surgical specimens from 30 patients with locally advanced head and squamous cell carcinoma (HNSCC) obtained prior to treatment (baseline), and after therapy (post-treatment). Tumor-immune microenvironmental changes and treatment outcomes were comparatively analyzed by stratifying baseline profiles according to immune cell density and composition. **B** Intratumoral cell densities (cells/mm^2^) of ten immune cell lineages were quantified, comparing baseline and post-treatment status after combination treatment with paclitaxel, carboplatin, and cetuximab. The classification of immune profiles into hypo-, lymphoid-, and myeloid-inflamed statuses was determined based on previously published cutoff values (see Materials and Methods). T_REG_ = regulatory T cells, and TAM = tumor-associated macrophages. **C** Representative longitudinal changes stratified by baseline immune profiles. Pan-cytokeratin (pCK), lymphoid lineages (CD3, CD20, and NKp46), and myeloid lineages (CD68, CD66b, DC-LAMP, and tryptase) are shown with indicated colors. Scale bar = 100 μm. **D** Violin plots for tumor volume, density of CD45^+^ cells, and combined positive score (CPS) of programmed death-ligand 1 (PD-L1), comparing baseline and post-treatment stratified by immune profiles. Bars represent the median and interquartile ranges. Statistical significance was determined via Wilcoxon matched-pairs signed-rank tests. **E**, **F** Kaplan–Meier analysis of relapse-free survival (RFS) (**E**) and overall survival (OS) (**F**), stratified by immune profiles. Statistical significance was determined via Gehan-Breslow-Wilcoxon tests.

To elucidate the alterations in the tumor-immune microenvironment in response to chemotherapy and targeted therapy, we comparatively analyzed the clinical outcomes and immune profiles of tumor tissues before and after treatment. A cohort of 30 patients with advanced HNSCC underwent combination therapy with paclitaxel, carboplatin, and cetuximab as preoperative chemotherapy. Biopsy specimens obtained prior to treatment served as the baseline, and surgically excised specimens obtained following therapy were considered post-treatment (Fig. 1A). A 14-marker multiplex IHC was used to quantitatively assess changes in the tumor-immune microenvironment, enabling the stratification of specimens based on the density and composition of 10 representative immune cell lineages. The three immune profiles identified in our previous study^2^ were observed in this cohort. The hypo-inflamed group featured few immune cells (1500 cells/mm^2^). In the lymphoid-inflamed group, lymphoid cells outnumbered myeloid cells by more than two-fold. In the myeloid-inflamed group, myeloid cells comprise more than half of the lymphoid cells (see Materials and methods) (Fig. 1B and C). Comparative analysis between baseline and post-treatment revealed that immune cell densities changed after combination therapy (Fig. 1B and C). Notably, tumors that were initially classified as hypo-inflamed transitioned to profiles characterized by high immune cell infiltration, whereas those in the myeloid-inflamed group largely maintained their original immune profiles (Fig. 1B), suggesting that the immune microenvironmental alterations during combination therapy might depend on baseline immune profiles.

Tumor volume, density of CD45^+^ cells, and combined positive score (CPS) defined as the number of programmed cell death ligand 1 (PD-L1)-positive cells (tumor cells, lymphocytes, and macrophages)^7^ were statistically analyzed before and following therapy. A significant decrease in tumor volume was observed in the hypo and lymphoid-inflamed groups, accompanied by a significant increase in the density of CD45^+^ cells and CPS (Fig. 1D). Conversely, the myeloid-inflamed group exhibited minimal changes in tumor volume, CD45^+^ cells, and CPS (Fig. 1D). Additionally, the myeloid-inflamed group showed a significantly shorter recurrence-free survival and a trend towards shorter overall survival, indicating a poorer prognosis (Fig. 1E and F). These findings suggest that the hypo-inflamed and lymphoid-inflamed groups responded to combination therapy, potentially undergoing significant immunological changes, whereas the myeloid-inflamed group exhibited a limited response.

### Elevation of cell death-related markers and lymphoid immune cells following combination therapy

**Fig. 2:**
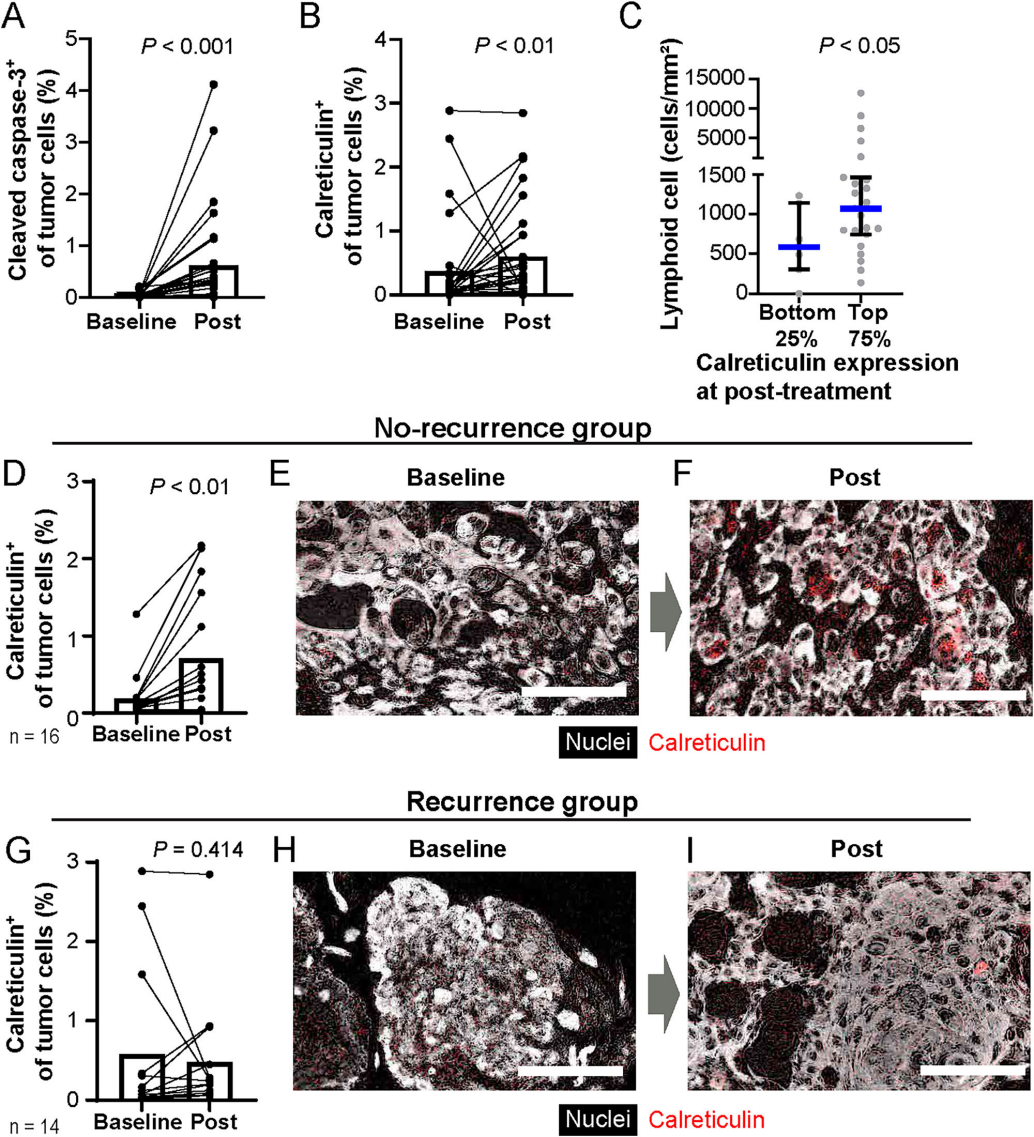
Cell death-related markers associated with changes in immune cell density and treatment outcomes during chemo-targeted therapy. **A, B** Percentages of cleaved caspase-3^+^ (**A**) and calreticulin^+^ (**B**) tumor cells at baseline and following treatment (*N* = 30). Bars represent the median. **C** Cell density of lymphoid cells following treatment stratified by post-treatment calreticulin expression levels. Statistical difference was determined using the Mann-Whitney U test. **D** Calreticulin^+^ cells among total tumor cells were quantified, comparing baseline and post-treatment status in the non-recurrence group. Bars represent the median. **E**, **F** Images showing expression of calreticulin at baseline (**E**) and post-treatment (**F**). Colors are shown. Scale bar = 100 μm. **G** Calreticulin^+^ cells among total tumor cells were quantified, comparing baseline and post-treatment status in the recurrence group. Bars represent the median. **H**, **I** Images showing expression of calreticulin from baseline (**H**) and post-treatment (**I**). Colors are shown. Scale bar = 100 μm. Statistical differences in (**A**, **B** and **D**–**I**) were determined via Wilcoxon matched-pairs signed-rank tests.

Given the reported induction of immunogenic cell death by chemotherapeutics, including taxanes^8^, we analyzed markers related to cell death, including cleaved caspase 3 for apoptosis^9^ and calreticulin for immunogenicity^10–12^. Cleaved caspase 3 levels increased in cancer cells, suggesting the promotion of apoptosis by the combination therapy (Fig. 2A and Supplementary Fig. 1A−C). Notably, calreticulin expression, which is associated with immunogenic cell death^9^, was also significantly increased in cancer cells after treatment (Fig. 2B). In particular, specimens exhibiting high calreticulin expression after treatment demonstrated an elevated density of lymphoid cells (Fig. 2C). Furthermore, a significant increase in calreticulin levels was observed after treatment in the group without recurrence (Fig. 2D and F), whereas no significant difference was noted in the group that experienced recurrence (Fig. 2G and I). These results suggest that combination therapy may enhance immunogenic cell death and subsequent lymphoid cell infiltration.

### Myeloid-inflamed status is associated with a tumor-promoting environment in relation to potential therapeutic resistance

**Fig. 3:**
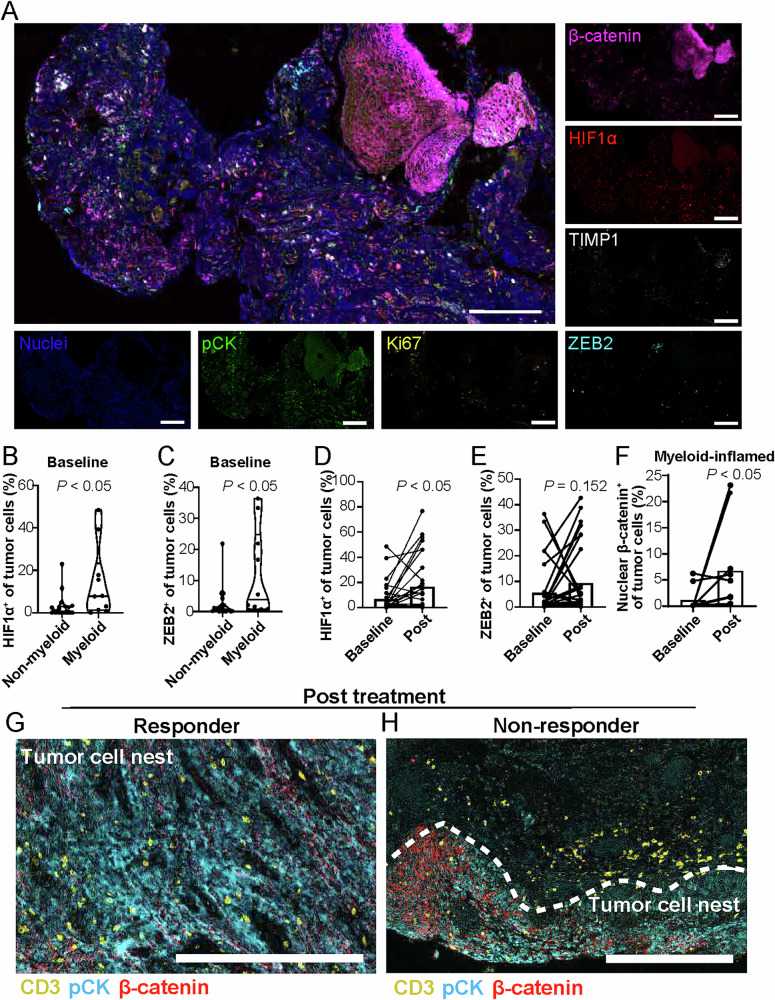
Myeloid-inflamed tumors feature high expression of hypoxia and metastasis-promoting factors, and spatial exclusion of T cells. **A** Representative image of myeloid-inflamed tissue visualized by biomarkers, including hypoxia inducible factor-1α (HIF1α), zinc finger E-box-binding homeobox-2 (ZEB2), β-catenin, Ki67, tissue inhibitor of metalloproteinases-1 (TIMP1), and pan-cytokeratin (pCK) with indicated colors. Scale bar = 100 μm. **B**, **C** Percentages of HIF1α^+^ (**B**) and ZEB2^+^ (**C**) tumor cells, comparing the myeloid- and non-myeloid-inflamed groups at baseline. Bars represent the median and interquartile ranges. Statistical difference was determined via Mann-Whitney U test. **D**, **E** Percentages of HIF1α^+^ (**D**), and ZEB2^+^ (**E**) of tumor cells, comparing baseline and post-treatment status (*N* = 30). **F** Percentages of nuclear β-catenin^+^ of tumor cells in the myeloid-inflamed group, comparing baseline and post-treatment status (*n* = 10). Statistical differences in (**D**–**F**) were determined via Wilcoxon matched-pairs signed-rank tests. Bar graphs represent mean values. **G**, **H** Images of representative post-treatment tissue from a responder (**G**) and a non-responder (**H**). Biomarkers and colors are shown. Scale bar = 300 μm.

Cancer tissues with a high proportion of myeloid cells exhibit therapeutic resistance^13–15^. Accordingly, we further analyzed the tumor microenvironmental characteristics. The multiplex IHC panel that was used included hypoxia inducible factor-1α (HIF1α), zinc finger E-box-binding homeobox-2 (ZEB2), β-catenin, Ki67, and tissue inhibitor of metalloproteinases-1 (TIMP1), which were specifically designed to target biomarkers associated with poor prognosis in HNSCC^15^ (Fig. 3A). Quantitative baseline evaluation revealed significantly higher expression of HIF1α and ZEB2 in the myeloid-inflamed group than the other groups (Fig. 3B and C), suggesting the presence of the hypoxic environment represented by HIF1α and the metastasis-promoting characteristics indicated by ZEB2 in the myeloid group. Furthermore, HIF1α and ZEB2 expression levels appeared to increase post-treatment (Fig. 3D and E), with a similar trend observed in the myeloid-inflamed group (Supplementary Fig. 2A and B). TIMP1, which is associated with a poor prognosis in head and neck cancer^15^, exhibited significantly increased expression in cancer cells after treatment (Supplementary Fig. 2C). β-catenin, which is involved in immune evasion and is associated with a poor prognosis^15,16^, exhibited significantly upregulated expression in the nuclei of cancer cells exclusively in the myeloid-inflamed group (Fig. 3F and Supplementary Fig. 2D). These findings suggest that the therapeutic resistance observed in the myeloid-inflamed group may be influenced by hypoxic and metastasis-promoting environments, indicating potential interactions between the tumor microenvironment and the inherent properties of cancer cells in determining therapeutic outcomes.

### Spatial status of the residual tumor microenvironment following combination therapy

As the distribution of intratumoral cellular components within the residual tumor microenvironment may offer insights into treatment resistance, we next focused on the spatial profiles of the tumor-immune microenvironment following combination therapy. Responders displayed abundant T cells in the tumor cell nest and surrounding tumor area (Fig. 3G). In non-responders, T cells were less abundant within the tumor cell nests compared to their surroundings. This tendency was particularly pronounced when β-catenin was expressed in the tumor cell nests (Fig. 3H). These findings suggest that residual tumors after treatment may exhibit resistance to T cell infiltration, potentially facilitated by β-catenin expression, which appears to spatially restrict intratumoral immune cell infiltration.

### Longitudinal analysis of immune checkpoint molecule expression in response to combination therapy

**Fig. 4:**
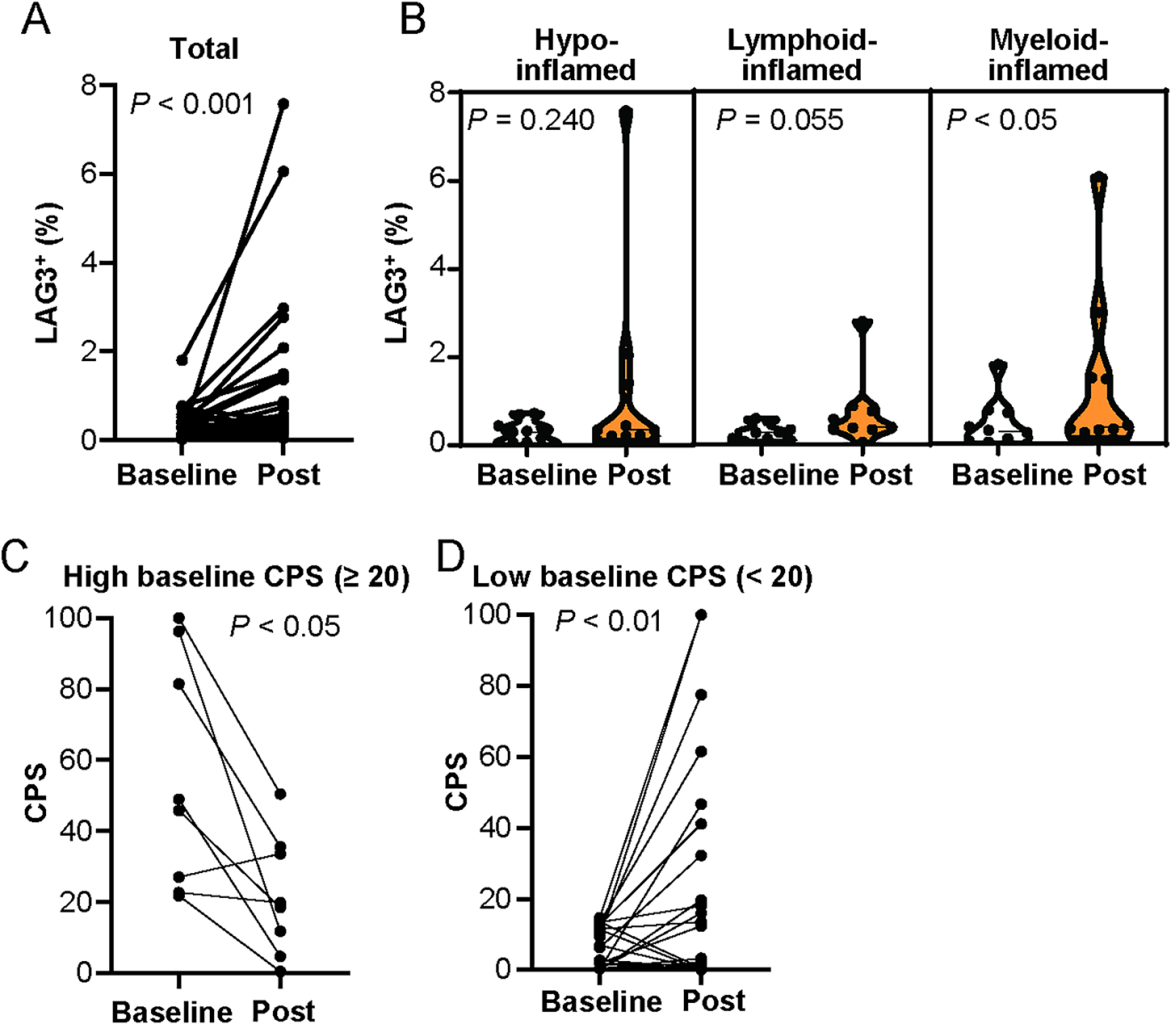
Longitudinal analysis of LAG3 and PD-L1 expression in the patients with HNSCC undergoing chemo-targeted therapy. **A** Percentages of LAG3^+^ non-tumor cells (pCK^–^) at baseline and post-treatment (*N* = 30). Statistical significances were determined via Wilcoxon matched-pairs signed-rank tests. **B** Violin plots showing the percentages of LAG3^+^ non-tumor cells (pCK^–^), comparing baseline and post-treatment, stratified by immune profiles. Dots in the violin plots represent individual cases, and vertical bars represent the median, and interquartile ranges. Statistical differences were determined via Wilcoxon signed-rank tests. **C**, **D** Longitudinal changes in the combined positive score (CPS) of PD-L1, comparing high baseline CPS ( ≥ 20) (**C**) and low baseline CPS ( < 20) (**D**). Statistical differences were determined via Wilcoxon signed-rank tests.

Understanding the status and dynamics of immune checkpoint molecules is essential to identify potential targets for subsequent or combination strategies, especially in treatment-resistant subgroups. Lymphocyte activation gene 3 (LAG3), an inhibitory receptor that is expressed on exhausted T cells and is a target for combinational immunotherapy^17^, increased significantly after treatment (Fig. 4A), particularly in the myeloid-inflamed group (Fig. 4B). These observations indicate that LAG3 may be a target for subsequent or combination treatments in the resistant myeloid-inflamed group. The dynamics of CPS with the expression status of PD-L1 expression status revealed a divergent pattern before and after treatment (Fig. 4C and D). Tumors with a high baseline CPS (≥20) experienced a significant decrease in CPS following the combination therapy (Fig. 4C), whereas those with a low baseline CPS (<20) exhibited a significant increase in CPS post-treatment (Fig. 4D). These findings suggest that combination therapy modulates the density and spatial distribution of immune cells, and alters their functional status, as evidenced by the observed dynamics in immune checkpoint molecule expression.

## Discussion

The present investigation of HNSCC utilized a multiplex IHC profiling platform to analyze the tumor-immune microenvironment before and after chemo- and targeted therapy. The findings highlight the treatment-induced dynamics of immune cell frequency, distribution, and spatial status, emphasizing the possibility of an interventional strategy for tumor-immune microenvironment-stratified optimal treatment.

Density, composition, distribution, and functional status are strongly associated with tumor characteristics. The predominance of lymphoid cells correlates with a favorable prognosis, whereas myeloid cell dominance suggests a poor prognosis^18^. Our results revealed that chemo- and targeted therapy positively affected both lymphoid-inflamed and immunologically cold tumors. To identify the potential mechanisms underlying the favorable response in hypo-inflamed tumors, cell death-related biomarkers were explored. In agreement with previous reports demonstrating the immunogenicity of cetuximab and taxanes such as paclitaxel^4,5,19^, we observed elevated expression of calreticulin following treatment together with increased lymphoid immune cell densities and low recurrence rate (Fig. 2D and F), suggesting the involvement of immunogenic cell death by the combination treatment.

Myeloid cells, such as tumor-associated macrophages, granulocytes, and mast cells, are involved in tumor progression and therapeutic resistance through immunosuppression, altered metabolism, and angiogenesis^14^. Our results support the association between myeloid cells and therapeutic resistance, where elevated expression of HIF1α and ZEB2 were observed in the myeloid-inflamed group, potentially related to an immunosuppressive and metastasis-promoting environment. HIF1α is a critical component in the cellular response to a hypoxic environment^20^, suppressing T cell function via myeloid cell-mediated mechanisms^21^. ZEB2 is associated with epithelial-to-mesenchymal transition, which is associated with immunosuppression by M2-like macrophages and regulatory T cells^22^. Together, these observations suggest that myeloid-inflamed tumors require additional treatment strategies by targeting myeloid cell populations and microenvironmental factors.

The interaction between tumor and immune cells and their dynamics during treatment can provide an in-depth understanding of the factors related to therapeutic response and resistance. β-catenin pathway activation is considered to exclude T cells from tumor cell nests, resulting in the establishment of a resistant tumor-immune microenvironment^23,24^. The spatial relationship between the tumor and immune cells within the residual tumor identified in this study may be related to the reduction in immune-mediated mechanisms via chemo- and targeted therapies as a possible indicator of poor response to treatment. In addition, although our investigation revealed treatment-induced modulation of the CPS, LAG3, and β-catenin, we did not detect statistically significant associations between either pre- or post-treatment values of these markers and patient survival or tumor recurrence. While this lack of prognostic significance could be viewed as a limitation of our study, it also suggests that the dynamic changes in these immune markers might provide valuable insights into the optimal timing for interventions with immune checkpoint inhibitors such as PD-1 blockade and potential LAG3-targeted therapies into neoadjuvant treatment strategies^25^. Moreover, these findings indicate that further studies involving larger cohorts and extended follow-up are warranted to fully delineate the clinical utility of these markers as predictive tools for therapeutic stratification.

In conclusion, our study underscores the significance of baseline immune characteristics in stratifying tumors into distinct categories based on the composition of immune cells. Building on our previous study^2^, which primarily focused on pre-treatment immune profiles, the present work extends these earlier findings by incorporating detailed spatial profiling and longitudinal analysis of treatment-induced immune alterations. These classifications have profound implications for clinical outcomes, including the response to chemo- and targeted therapies. The development of this framework facilitates the selection of optimized treatments tailored to individual patients and provides strategies for subsequent therapeutic choices following the initial treatment. Finally, this approach fosters the personalization of treatment regimens, enabling the selection of the most effective combination of therapies, considering each patient’s tumor-immune microenvironmental properties. This advancement represents a step toward tissue-based personalized medicine in the field of head and neck cancer.

## Methods

### Ethics approval and consent to participate

All studies involving human tissue were approved by the institutional review board of Kyoto Prefectural University of Medicine (ERB-C-43-4), and written informed consent was obtained. All studies involving human research participants, material, or data have been performed in accordance with the Declaration of Helsinki.

### Patients, and clinical evaluations

This study included 30 patients with locally advanced HNSCC who were diagnosed and treated at Kyoto Prefectural University of Medicine. All patients received a preoperative combination therapy regimen consisting of paclitaxel, carboplatin, and cetuximab. The treatment protocol involved the administration of paclitaxel (100 mg/m²) and carboplatin (AUC 2) weekly for two weeks followed by a one-week break, and cetuximab at an initial dose of 400 mg/m², followed by a weekly dose of 250 mg/m². Surgical resection was performed after completion of the six-week combination therapy regimen. Biopsy specimens were obtained prior to the initiation of therapy to serve as baseline samples, and surgically excised specimens were collected following therapy to assess treatment-induced changes. Detailed clinicopathological characteristics of the patients are presented in Table 1. Baseline and post-treatment CT or MRI scans were utilized to evaluate treatment response in accordance with RECIST v1.1 criteria. Responders were defined as patients with complete response or partial response, while non-responders were classified as those with stable disease or progressive disease. Tumor volume was calculated using the formula: V = A × B^2^/2, where A and B represent the long and short axes of the tumor, respectively.

**Table 1:** Clinical characteristics of patients enrolled in this study

		Responder	Non-responder	
		*n* = 21	*n* = 9	*P* value
Sex	Female	9	1	0.2035
	Male	12	8	
Age	65 <	8	5	0.4434
	65 ≥	13	4	
T stage	1 & 2	4	4	0.1954
	3 & 4	17	5	
N stage	0	14	5	0.6871
	1 & 2	7	4	
M stage	0	21	9	> 0.9999
	1	0	0	
Stage	I-II	3	3	0.3287
	III-IV	18	6	
Site	Oral	12	4	0.4542
	Oropharynx	4	4	
	Hypopharynx	2	0	
	Larynx	3	1	
p16 status	Negative	2	0	0.4643
	Positive	3	3	
	Unknown	16	6	
Surgical margin	Negative	16	5	0.3888
	Positive	5	4	
Postoperative	Absence	17	6	0.6402
radiation therapy	Presence	4	3	

### Multiplex IHC

Multiplex IHC was performed using a previously described method^2^. Briefly, following deparaffinization and blocking of peroxidase activity, formalin-fixed paraffin-embedded sections 5 μm in thickness were subjected to sequential IHC with validated antibodies based on cycles of staining, scanning, and antibody/chromogen stripping. After chromogen development to revealed the binding sites of the antibodies, the slides were scanned digitally at an objective magnification of 20× using a Nanozoomer S60 scanner (Hamamatsu Photonics, Shizuoka, Japan). The conditions and antibodies used in this study are listed in Supplementary Tables 1−3.

### Digital image processing and image cytometry

After staining, image acquisition and computational processing were performed using a previously described method^2^. For image preprocessing, iteratively digitized images were co-registered using in-house software (SCREEN Holdings Co., Ltd., Kyoto, Japan), which calculated the coordinates of each image relative to the reference hematoxylin-stained image. The co-registered images were converted to single-marker images, inverted, and converted to grayscale, followed by pseudo-coloring using ImageJ/Fiji Version 1.51 s (National Institutes of Health, Bethesda, MD, USA). Single-cell segmentation and quantification of staining intensity and shaped-size measurements were performed using CellProfiler Version 2.2.0 (Broad Institute, Cambridge, MA, USA) and saved in a file format compatible with FCS Express 7 Image Cytometry data analysis software (De Novo Software, Pasadena, CA, USA). A sequential gating strategy using image cytometry was used to identify cell phenotypes based on negative cell staining (Supplementary Table 4). Visualization was performed using Aperio ImageScope Version 12.3.3.5048 software (Leica, Wetzlar, Germany).

For imaging analysis, three regions of interest were selected from each image, representing the areas with the highest CD45^+^ cell density within a 2 × 2 mm² region. Based on cutoff values obtained from a previous study^2^, the tissue immune characteristics of the three groups of hypo-, lymphoid-, and myeloid-inflamed status were classified. If there were fewer than 1500 CD45^+^ cells/mm^2^, the specimen was classified as hypo-inflamed. If the lymphoid-to-myeloid cell ratio was >2, the specimen was classified as lymphoid-inflamed. If the ratio was <2, the specimen was classified as myeloid-inflamed. Nuclear β-catenin expression was quantified using ImageJ/FIJI by extracting and quantifying β-catenin expression pixels overlapping with hematoxylin.

### Statistical analyses

Statistical analyses were performed using the GraphPad Prism 8.3.0 (Graphpad Software, San Diego, CA, USA). Wilcoxon matched-pair signed-rank tests were used to compare paired samples. Mann–Whitney U tests were used for comparisons between unpaired groups. Chi-square tests or Fisher’s exact tests were used for clinicopathological factors. Overall and relapse-free survival were estimated using the Kaplan–Meier method, and the Gehan–Breslow–Wilcoxon test was used for statistical significance analysis. Statistical significance was set at p < 0.05.

## Supplementary information

Supplementary Material

## Data Availability

The data that support the findings of this study are available from the corresponding author upon reasonable request.

## Code availability

All relevant codes are available from the corresponding authors upon reasonable request.
