# Supplementary material for: Neoadjuvant therapy-induced immune dynamics and myeloid-associated resistance in advanced head and neck cancer
Source: NPJ Precis Oncol. 2025 Jun 7;9:167. doi: 10.1038/s41698-025-00954-1 (PMC12145449; doi:10.1038/s41698-025-00954-1)
Supplement: Supplementary file 1 — Supplementary Material [file 41698_2025_954_MOESM1_ESM.pdf]

Supplementary Material

Immune microenvironmental alternations related to efficacy and resistance for chemo- and targeted therapy in head and neck squamous cell carcinoma

Authors: Alisa Kimura, Junichi Mitsuda, Kanako Yoshimura, Sumiyo Saburi, Nanako Murakami, Nana Sakurai, Koichi Yoshizawa, Hiroki Morimoto, Shigeyuki Mukudai, Hikaru Nagao, Hiroshi Ogi, Saya Shibata, Aya Miyagawa-Hayashino, Eiichi Konishi, Kyoko Itoh, Shigeru Hirano, Takahiro Tsujikawa

Supplementary Figure 1. Cleaved caspase 3 expression during chemo-targeted therapy.

Supplementary Figure 2. Tumor microenvironmental markers during chemo-targeted therapy.

Supplementary Table 1. A list of antibodies and conditions used for immune composition panel.

Supplementary Table 2. A list of antibodies and conditions used for tumor microenvironmental marker panel.

Supplementary Table 3. A list of antibodies and conditions used for cell death marker panel.

Supplementary Table 4. Identification markers for immune cell lineages.

Supplementary Figure 1

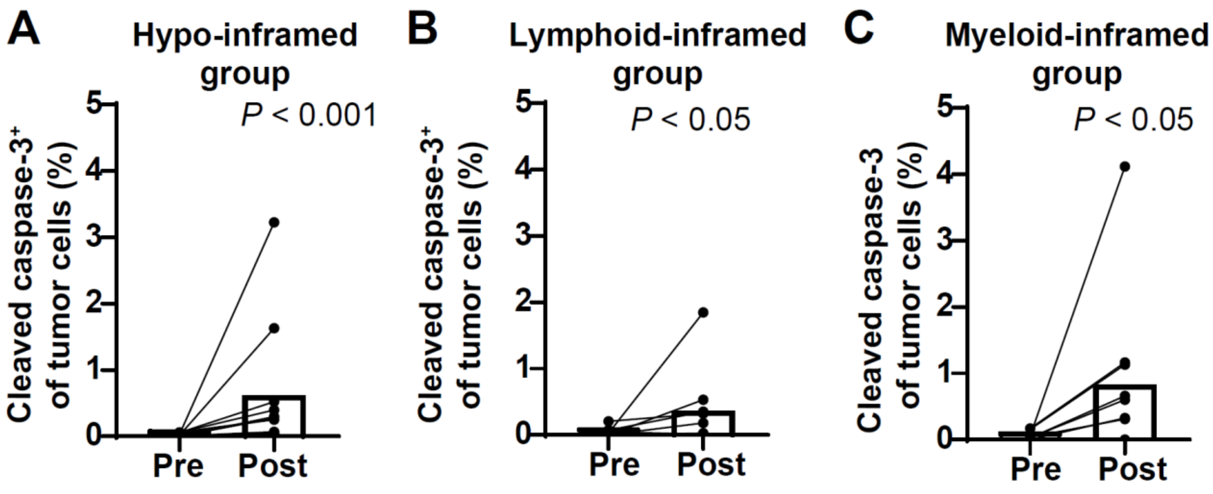

Supplementary Figure 1. Cleaved caspase 3 expression during chemo-targeted therapy.

(**A−C**) Percentages of cleaved caspase-3^{+} tumor cells at baseline and post-treatment status were comparatively evaluated, stratified into the hypo-inflamed (n = 11) (A), lymphoid-inflamed (n = 9) (B), and myeloid-inflamed groups (n = 10) (C). Bars represent the median. Statistical differences were determined via Wilcoxon matched-pairs signed rank tests.

Supplementary Figure 2

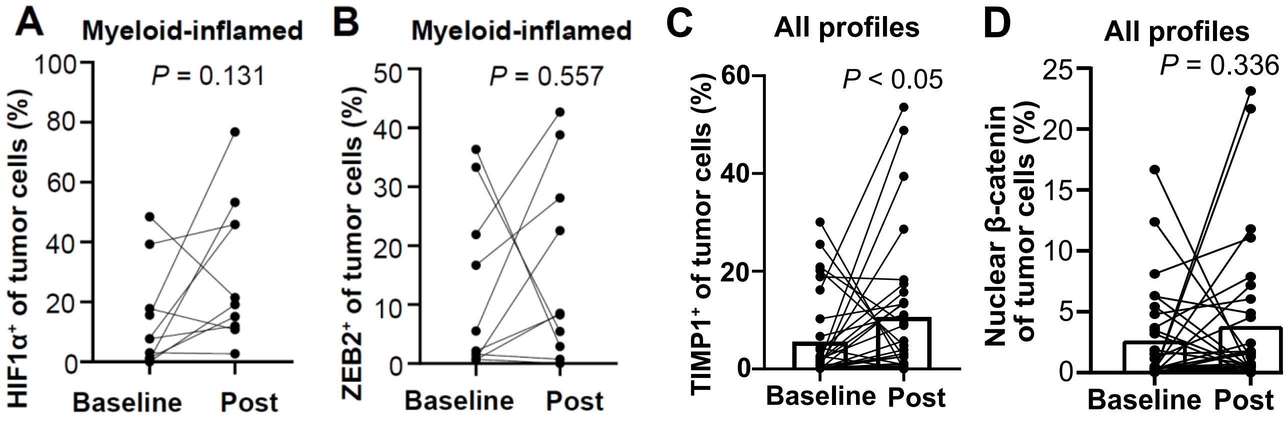

Supplementary Figure 2. Tumor microenvironmental markers during chemo-targeted therapy.

(**A–B**) Percentages of HIF1α^{+} (A) and ZEB2^{+} (B) tumor cells in the myeloid-inflamed group, comparing baseline and post-treatment status (n = 10). (**C–D**) Percentages of TIMP1^{+} (C) and nuclear β-catenin^{+} (D) tumor cells in the all profiles, comparing baseline and post-treatment status (N = 30). Statistical differences were determined via Wilcoxon matched-pairs signed-rank tests.

Supplementary Table 1. A list of antibodies and conditions used for immune composition panel.

	Cycle1	Cycle2	Cycle3	Cycle4	Cycle5	Cycle6	Cycle7
Primary Ab	Hematoxylin	PD1	CD8	CD3	NKp46	CD68	CD45
Clone/Product#	S3301	NAT105	C8/144B	SP7	195314	PG-M1	H130
Supplier	Dako	Abcam	Thermo science	Thermo science	R&D	Abcam	Thermo science
Concentration		1/50	1/100	1/50	1/200	1/50	1/100
Reaction	2min	RT, 30min	RT, 30min	RT, 30min	RT, 30min	RT, 30min	RT, 30min
Secondary Ab		Anti-Mouse	Anti-Mouse	Anti-rabbit	Anti-Mouse	Anti-Mouse	Anti-Mouse
Reaction		RT, 30min	RT, 30min	RT, 30min	RT, 30min	RT, 30min	RT, 30min
AEC			20min		20min	20min	20min
AMEC		5min		5min			

	Cycle8	Cycle9	Cycle10	Cycle11	Cycle12	Cycle13	Cycle14
Primary Ab	PDL1	DC-LAMP	Foxp3	CD20	CD66b	Tryptase	Pan-CK
Clone/Product #	EIL3N	16H11.2	236A/E7	L26	G10F5	AA1	AE1/AE3
Supplier	Cell signaling	EMD Millipore	eBioscience	Abcam	eBioscience	Abcam	Abcam
Concentration	1/100	1/200	1/40	1/200	1/400	1/20,000	1/2000
Reaction	4°C overnight	RT, 30min	RT, 30min	RT, 30min	RT, 30min	RT, 30min	RT, 30min
Secondary Ab	Anti-rabbit	Anti-Mouse	Anti-Mouse	Anti-Mouse	Anti-Mouse	Anti-Mouse	Anti-Mouse
Reaction	RT, 30min	RT, 30min	RT, 30min	RT, 30min	RT, 30min	RT, 30min	RT, 30min
AEC		20min	20min	20min	20min	20min	20min
AMEC	5min						

AEC: AEC Substrate Kit, Peroxidase (HRP), (3-amino-9-ethylcarbazole) (SK-4200)
AMEC: ImmPACT® AMEC Red Substrate Kit, Peroxidase (HRP) (SK-4285)

Supplementary Table 2. A list of antibodies and conditions used for tumor microenvironmental marker panel.

	Cycle1	Cycle2	Cycle3	Cycle4	Cycle5	Cycle6	Cycle7
Primary Ab	Hematoxylin	ZEB2	HIF1α	β-catenin	CD3	DKK-1	PINCH1
Clone/Product#	S3301	HPA003456	ab114977	ab16051	SP7	ab61034	MABT162
Supplier	Dako	Merck	Abcam	Abcam	Thermo science	Abcam	Merck
Concentration		1/75	1/75	1/300	1/100	1/100	1/40
Reaction time	2min	RT, 30min	RT, 30min	RT, 30min	RT, 30min	RT, 30min	RT, 30min
Secondary Ab		Anti-rabbit	Anti-rabbit	Anti-rabbit	Anti-rabbit	Anti-rabbit	Anti-mouse
Reaction time		RT, 30min	RT, 30min	RT, 30min	RT, 30min	RT, 30min	RT, 30min
AEC		20min	20min	20min	20min	20min	20min

	Cycle8	Cycle9	Cycle10	Cycle11	Cycle12	Cycle13	Cycle14
Primary Ab	α-SMA	CD68	ZFX	ADAM10	Ki-67	pCK	TIMP1
Clone/Product#	ab5694	PG-M1	HPA003877	ab19026	SP6	AE1/AE3	ab211926
Supplier	Abcam	Abcam	Merck	Merck	Sigma-Aldrich	Abcam	Abcam
Concentration	1/200	1/50	1/1200	1/2000	1/2000	1/2000	1/1000
Reaction time	RT, 30min	RT, 30min	RT, 30min	RT, 30min	RT, 30min	RT, 30min	RT, 30min
Secondary Ab	Anti-rabbit	Anti-mouse	Anti-rabbit	Anti-rabbit	Anti-rabbit	Anti-mouse	Anti- rabbit
Reaction time	RT, 30min	RT, 30min	RT, 30min	RT, 30min	RT, 30min	RT, 30min	RT, 30min
AEC	20min	20min	20min	20min	20min	20min	20min

Supplementary Table 3. A list of antibodies and conditions used for cell death marker panel.

	Cycle1	Cycle2	Cycle3	Cycle4	Cycle5	Cycle6
Primary Ab	Hematoxylin	CCP3	CALR	Ki-67	PanCK	LAG3
Clone/Product#	Dako	ASP175	FMC75	SP6	AE1/AE3	EPR4392
Supplier		Cell signaling	Abcam	Sigma-Aldrich	Abcam	Abcam
Concentration	Original	1/400	1/20000	1/100	1/2000	1/1000
Reaction	2min	4°C overnight	RT, 30min	RT, 30min	RT, 30min	30min, RT
Secondary Ab		Anti-rabbit	Anti-Mouse	Anti-rabbit	Anti-Mouse	Anti-rabbit
Reaction		30min, RT	30min, RT	30min, RT	30min, RT	30min, RT
AEC		20min	20min	20min	20min	20min

Supplementary Table 4. Identification markers for immune cell lineages.

Immune cell subpopulation	Abbreviation	Marker
$CD8^{+}$ T cells	CD8 T	$CD45^{+}$ $CD3^{+}$ $CD8^{+}$
Regulatory T cells	T_{REG}	$CD45^{+}$ $CD3^{+}$ $CD8^{-}$ $Foxp3^{+}$
Helper T cells	Helper T	$CD45^{+}$ $CD3^{+}$ $CD8^{-}$ $Foxp3^{-}$
B cells	B cell	$CD45^{+}$ $CD3^{-}$ $NKp46^{-}$ $CD20^{+}$
Natural killer cells	NK	$CD45^{+}$ $CD3^{-}$ $NKp46^{+}$
Mast cells	Mast cell	$CD45^{+}$ $CD3^{-}$ $CD20^{-}$ $CD66b^{-}$ $Tryptase^{+}$
Granulocytes	Gr	$CD45^{+}$ $CD3^{-}$ $CD20^{-}$ $CD66b^{+}$
Tumor associated macrophages	TAM	$CD45^{+}$ $CD3^{-}$ $CD20^{-}$ $CD66b^{-}$ $Tryptase^{-}$ $CD68^{+}$
Dendritic cells	DC	$CD45^{+}$ $CD3^{-}$ $CD20^{-}$ $CD66b^{-}$ $Tryptase^{-}$ $CD68^{-}$ $DC\text{-}LAMP^{+}$
$CD45^{+}$ other	$CD45^{+}$ other	$CD45^{+}$ $CD3^{-}$ $CD20^{-}$ $CD66b^{-}$ $Tryptase^{-}$ $CD68^{-}$ $DC\text{-}LAMP^{-}$
